# Clinical development of IL13Rα2-targeting CAR T cells for the treatment of glioblastoma

**DOI:** 10.1186/2051-1426-3-S2-P114

**Published:** 2015-11-04

**Authors:** Christine E Brown, Renate Starr, Brenda Aguilar, Alfonso Brito, Brenda Chang, Aniee Sarkissian, Lihong Weng, Michael Jensen, Michael E Barish, Behnam Badie, Stephen J Forman

**Affiliations:** 1 Beckman Research Institute, City of Hope National Medical Center, Duarte, CA, USA; 2 Seattle Children's Research Institute, Seattle, WA, USA

## 

T cell immunotherapy is emerging as a powerful strategy to treat cancer, and may offer new opportunities to improve outcomes for patients with glioblastoma (GBM). Our group has developed a chimeric antigen receptor (CAR) T cell immunotherapy for GBM targeting IL-13 receptor α2 (IL13Rα2), a cell surface receptor over-expressed by the majority of high-grade gliomas. Towards this end, we have optimized IL13Rα2-specific CAR T cells by incorporating enhancements in CAR design and T cell engineering to improve T cell persistence and antitumor potency. These include a second-generation CAR containing the 41BB (CD137) costimulatory signaling domain (IL13BBζ-CAR), and a manufacturing strategy using an enriched central memory T cell (T_CM_) population for genetic engineering. To model clinical conditions and translate this therapy to patients we used orthotopic human GBM models with low-passage patient-derived tumor sphere (TS) lines in NSG mice. We demonstrate here that single injections of optimized IL13BBζ-CAR T_CM_ results in significantly improved antitumor activity and T cell persistence as compared to the early-generation IL13Rα2-CAR T cells that were themselves capable of mediating transient anti-tumor activity in patients. Evaluating routes for delivery, intravenous (i.v.) versus intracranial (i.c.), we find that i.c. delivery of the therapeutic CAR T cells elicits superior antitumor efficacy as compared to i.v. administration, which in these studies provided no apparent therapeutic benefit. We also explored the capacity of T cells to traffic within the brain parenchyma, and using a multifocal disease model establish that CAR T cells injected i.c. at one tumor site are able to traffic to a second tumor site in the contralateral hemisphere. Further, we investigated variations in cell product composition, including CD4 to CD8 ratios, and observed greater tumor recurrence in mice that received 100% CD8^+^ CAR T cells, indicating that inclusion of CD4+ CAR T cell subsets improves the durability of the therapeutic response. Finally, we evaluated the impact of corticosteroid, given its frequent use in clinical management of GBM, and demonstrate that low dose dexamethasone does not diminish T cell antitumor activity *in vivo*. These findings refine both CAR T cell product and clinical parameters for optimally translating this therapy to patients, and provide the rational for our newly initiated first-in-human phase I clinical trial evaluating intracranial administration of IL13Rα2-specific CD4^+^ CD8^+^ IL13BBζ-CAR T_CM_ for the treatment of GBM.

